# Reproducible analytical pipelines for healthcare discrete‑event simulation: An open guide and worked examples

**DOI:** 10.3310/nihropenres.14296.1

**Published:** 2026-06-16

**Authors:** Amy Heather, Thomas Monks, Alison Harper, Fatemeh Alidoost, Robert Challen, Thomas Slater, Navonil Mustafee

**Affiliations:** 1 Peninsula Collaboration for Health Operational Research and Data Science, University of Exeter Medical School, Exeter, England, UK; 2 Centre for Simulation, Analytics, and Modelling, University of Exeter Business School, Exeter, England, UK; 3 School of Engineering Mathematics and Technology, University of Bristol Faculty of Science and Engineering, Bristol, England, UK; 4 University of Exeter Department of Mathematics and Statistics, Exeter, England, UK

**Keywords:** Discrete-event simulation, healthcare, reproducibility, open science, open models

## Abstract

Discrete-event simulation (DES) is a popular technique for exploring problems in healthcare. For these models to be reused and have lasting impact, they need to be reproducible, transparent and well structured. Reproducible analytical pipeline (RAP) approaches are structured robust workflows that ensure analyses can be reproduced. They have emerged as best practice, but modellers struggle to implement them due to gaps in accessible guidance, skills, and time. This paper presents an integrated set of resources designed to help modellers bridge this implementation gap: an open-access e-book and four worked example repositories demonstrating complete RAP workflows for DES in both Python and R. The online book provides step-by-step guidance through nine major sections covering introductory material, project set-up, model inputs, model building, output analysis, experimentation, verification and validation, style and documentation, and collaboration and sharing. The case studies demonstrate varying complexity: a classic M/M/s queueing model, and a replication of a stroke care pathway model. The worked examples serve dual purposes: they demonstrate that RAP principles are achievable for healthcare DES models (from canonical queueing systems to real-world clinical pathways), and they provide templates that modelling teams can adopt and adapt within routine decision-support projects.

## Plain Language summary

This paper is about making it easier for people to build and share computer models that help improve healthcare services. These models are often used to look at things like how long patients wait and how busy staff and equipment are, but it can be hard for others to rerun or adapt them. We take ideas from “reproducible analytical pipelines”, which are tidy, fully documented ways of running analyses, and show how to use them for healthcare simulation. We created a free online book and four full examples in the Python and R programming languages that walk through every step: setting up a project, preparing data and parameters, writing and testing the model, and keeping the code style and documentation clear and consistent. All of this is open access, so people who want to create models can copy, learn from, and customise the materials for their own work.

## 1. Introduction

### 1.1. Discrete-event simulation in healthcare

Discrete-event simulation (DES) is a popular technique for exploring problems in healthcare, applied across a wide range of settings, including hospital and medical centres, emergency departments, and on patient clinical conditions. They have examined outcomes such as waiting times and other efficiency measures, the use and scheduling of resources, and impacts on costs ( Vázquez-Serrano et al., 2021).

While many DES studies achieve impact within the specific health systems they are designed to support, their wider value and impact depends on whether models and their results can be independently checked, built on, and adapted in new contexts. In computational science, this can be framed in terms of the “5 Rs” of good practice: that code and workflows should be re-runnable, repeatable, reproducible, reusable, and replicable ( Benureau and Rougier, 2018).

### 1.2. Reproducibility and reproducible analytical pipelines

In this paper, we focus on computational reproducibility, which is the ability of an independent researcher to regenerate the published results using the provided model code and data ( FORRT, 2026; Azevedo et al., 2019). DES are stochastic, so while controlling random seeds is useful for checking that the same code and seed reproduce the exact same results, Luijken et al. (2024) note that even without reported seeds, reproducibility can be assessed by showing that the average results across multiple replications remain consistent when the model is rerun with different seeds.

Reproducibility is important because it lets others verify results and trust that a model behaves as reported ( Sandve et al., 2013; Harper et al., 2021). Reproducibility is a prerequisite for safe reuse because only code that reliably regenerates published results can be confidently applied to new data or projects ( Nuijten et al., 2018). It also benefits authors, by making it easier to rerun, update, and extend their own analyses over time. Without reproducible code and environments, re-running analyses later (such as after peer review) can become slow or even impossible ( Alston and Rick, 2021).

A practical way to support reproducibility when modelling is to organise the analysis as a reproducible analytical pipeline (RAP). A RAP is an automated end-to-end workflow where every step can be run without manual intervention: from input modelling, to model execution, through to the creation of tables and figures. Beyond automation, RAPs embed software engineering best practices including version control, testing, code review, packaging and documentation ( Figure 1). The benefits of adopting a RAP approach are that it improves code quality, reduces manual steps, lowers the risk of errors, and helps to make the model and analysis more robust, sustainable and maintainable ( Analysis Function Central Team, 2025; Munro et al., 2023). In healthcare organisations, this maintainability is critical for operational DES models that must be reused, updated, and re-run as services, pathways, and data change over time.

**Figure 1.**
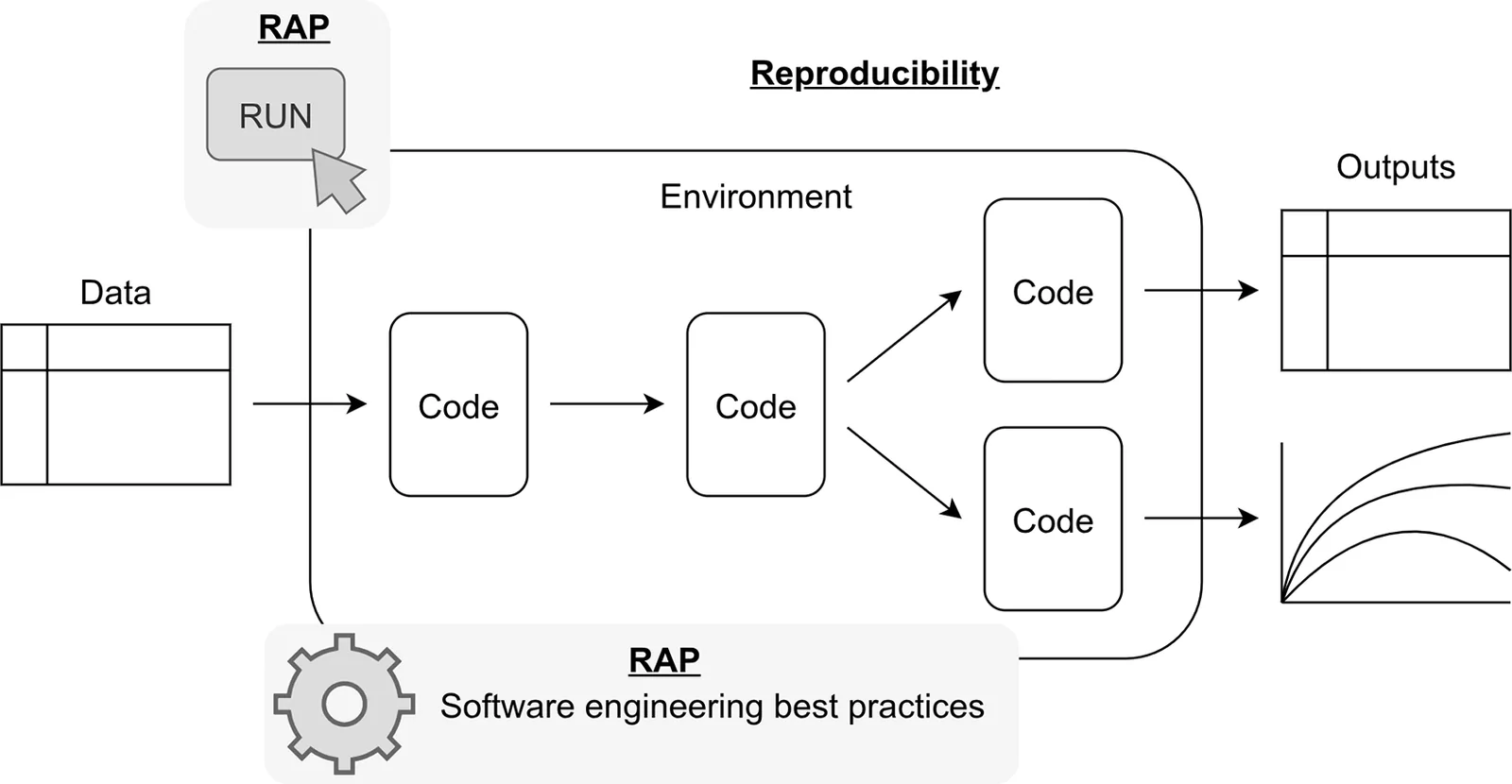
Reproducibility versus reproducible analytical pipelines (RAP).

### 1.3. Reproducibility of published simulation studies

Open surveys suggest that reproducibility problems are widespread across science: more than 70% of scientists have tried and failed to reproduce another group’s work and over half have failed to reproduce their own ( Baker (2016)). Simulation studies are no exception, as shown by several computational reproducibility assessments that include simulation models in their samples. In political science, an in-house review of 24 papers from the *Quarterly Journal of Political Science* found that only four ran easily without error, and that 58% produced results that differed from those reported, motivating recommendations such as clear README files, explicit specification of software dependencies, and setting and documenting random seeds ( Eubank, 2016). Similarly, in computational physics, attempts to reproduce seven articles with up to 40 hours of troubleshooting per study yielded only partial success in every case, with substantial effort spent resolving issues such as missing code or data, mismatches between the published methods and available scripts, and software or dependency conflicts ( Krafczyk et al., 2021).

However, other articles have found greater success. Fišar et al. (2024) assessed the reproducibility of articles in *Management Science* after the journal introduced a 2019 policy requiring authors to provide data and code; their sample included nearly 500 articles, of which about a fifth were simulation or other computational studies, while most reported empirical analyses based on observational or experimental data. Among the 297 articles for which all required datasets were accessible, 95.4% were fully or largely reproduced ( Fišar et al., 2024). Stodden et al. (2018) evaluated a random sample of 204 papers from *Science*, of which only 44% had any associated code or data. Many of these were then judged unlikely to be reproducible because of missing scripts, documentation, or key parameter settings, while 56 articles were considered potentially reproducible; from these, the authors selected 22 for detailed assessment and successfully reproduced the reported numerical results for all but one ( Stodden et al., 2018).

Two recent studies examine reproducibility in the context of healthcare simulations. Henderson et al. (2024) evaluated two samples of infectious disease modelling studies: one consisting of 100 randomly sampled papers and the other of the 100 most highly cited papers. For the random sample, the required code and data were only shared for 19 papers; of those, 4 were fully reproducible, 8 were partially reproducible, and 7 were not reproducible. For the highly cited sample, 48 papers shared all required materials; of these, 11 were fully reproducible, 22 were partially reproducible, and 15 were not reproducible ( Henderson et al., 2024).

In Heather et al. (2025), we conducted an in-depth assessment of eight published healthcare DES models implemented in Python or R, using a small but diverse sample to explore barriers and facilitators of reproduction in healthcare DES. Reproducing results required up to 28 hours of troubleshooting per model and, despite this substantial effort, only half were fully reproducible, while the remainder were only partially reproduced (12.5% to 94.1% of reported outcomes). Key barriers were mismatches between parameters described in the paper and those implemented in the code, missing scripts, and missing licences (meaning reuse was not clearly permitted until the paper authors added licences).

A systematic review of 182 open-source health economic models, of which 25% were simulation models, found that licensing and reuse conditions were uneven, with no evident licence for approximately a quarter of models ( Henderson et al., 2025) A recent scoping review looked at how healthcare DES models are shared across the published literature. Of the 564 studies identified, only 8.3% shared their model. Among those that did, sharing practices often fell short of supporting reproduction and reuse: licences were rarely included (only 37.5 to 48.4% had licenses), instructions on how to run the model were often missing or minimal, and few provided any form of dependency management or environment specification ( Monks and Harper, 2023). This suggests that limited and inconsistent sharing practices are a common barrier to making healthcare DES models reproducible and reusable. These challenges affect not only academic researchers but also analysts and modelling teams working in healthcare organisations like the NHS, who use DES to inform operational and strategic decisions.

### 1.4. Open science efforts in simulation and current gaps

There has been a sustained push towards better sharing and reporting of simulation studies. A panel at the 2016 Winter Simulation Conference argued that simulation models and code should be openly shared and highlighted the need for clearer standards for reporting simulation work ( Taylor et al., 2018). In response, guidelines have been developed, such as *Strengthening the reporting of empirical simulation studies* (STRESS) which specifies how studies should be reported to support replication ( Monks et al., 2019), and criteria to improve the reporting quality of DES studies from Zhang et al. (2020). Also, the *Sharing Tools and Artefacts for Reusable Simulations* (STARS) framework describes practical steps for facilitating reuse of DES models ( Monks et al., 2024).

To address the specific issue of reproducibility, Heather et al. (2025) propose a set of recommendations aimed at improving the reproducibility of healthcare DES models. Broader initiatives, such as The Open Modeling Foundation (2026)‘s minimal standards, and the *Levels of RAP* guidance developed by the NHS RAP Community of Practice (2025b), set out good practice, documentation requirements, and what is expected for RAP workflows. However, despite these resources providing a more concrete picture of reproducibility for simulation and of RAP, they focus on *what* to do rather than *how* to do it in practice.

Surveys across disciplines show that researchers broadly recognise the value of open, shared models but lack the practical knowledge and resources to deliver them. Pouwels et al. (2022) surveyed 230 members of the International Society for Pharmacoeconomics and Outcomes Research (ISPOR) about open-source health economic models. Most respondents agreed that open-source models would improve transparency (92%) and model reuse (86%). However, they reported several practical barriers: uncertainty about how models would be maintained and updated over time, legal and regulatory constraints, and the challenge of transferring underlying or related data in a secure way to permit model execution ( Pouwels et al., 2022). A survey of Wellcome Trust-funded researchers identified insufficient skills, time and funding to prepare code for sharing as the primary barriers ( Van den Eynden et al., 2016). In a survey of *PLOS Computational Biology* authors, code was not shared for nearly a third of their articles. Authors reported that lack of time to sufficiently prepare code for sharing was the most common reason, alongside concerns about their own ability to share code appropriately and about software and system dependencies ( Hrynaszkiewicz et al., 2021). A follow-up survey asked authors to rate their satisfaction with different aspects of code sharing and reuse, and being able to run code easily in the correct environment received the lowest scores. When asked how much extra time they would be willing to spend using a new tool to make their code easier to read and run, over half of respondents indicated a day or more, while others reported much tighter time constraints ( Cadwallader and Hrynaszkiewicz, 2022). More recently, Gelsleichter et al. (2025) found that 65% of researchers cited lack of time for proper documentation as a barrier to sharing code, while 55% identified training as the most important measure to overcome barriers to reproducibility. These findings highlight a persistent gap between the community’s desire for open models and its capacity to achieve them in practice.

Within healthcare organisations, there is likewise a gap between ambitions for open, reproducible analytics and everyday practice among analysts and development teams. A survey of NHS trusts reported that 73.9% of teams do not routinely use open-source approaches, and only 5.8% use modern version control or collaboration platforms like Git ( Bennion et al., 2025). National policy documents such as the Department of Health and Social Care’s independent review “ *Better, broader, safer: using health data for research and analysis*” set out best practice for NHS data analysis, emphasising the use of free and open-source tools, shared code, and reproducible analytical pipelines ( Goldacre et al., 2022), consistent with the NHS service standard requirement to make new source code open ( NHS England service manual team, 2026).

There are some practical resources on reproducibility such as The Turing Way (2025) which provides broad, step-by-step guidance for reproducible research, but this is intentionally generic and not tailored to DES. The *Little Book of DES* ( Rosser et al., 2025) also makes a valuable contribution by offering accessible tutorials for building simulation models in Python and touches on aspects of good practice. Although, its focus is on introducing DES methods rather than developing full reproducible workflows, and it only focuses on Python. As a result, there remains a major gap: the lack of comprehensive, end-to-end RAP workflows for DES, implemented in both Python and R, that show how to conduct reproducible healthcare DES studies and how to meet existing standards and recommendations in practice.

### 1.5. Objectives of this research

(1) To translate abstract reproducibility guidelines into concrete executable software examples.
(2) To directly tackle the “knowledge barrier” by providing a clear, gold standard, step-by-step process for modellers.
(3) To deliver a dual language implementation, standardised across Python and R, to maximise adoption and impact across the healthcare simulation community.

Having set out these objectives, we next describe the two main resources developed to achieve them: an open online book that provides a comprehensive RAP guide for DES, and a set of four worked example repositories that implement these principles in practice. Sections 3 and 4 outline the design and content of these resources. Section 6 then reflects on their implications, uses, and limitations.

## 2. Patient and public involvement

There was no patient and public involvement in this research.

## 3. Open online book on RAP for DES

### 3.1. Tools and infrastructure

We developed all examples in parallel in both Python and R, selected as the most widely used free and open-source options for healthcare DES ( Monks and Harper, 2023). The simulation logic was implemented using the most popular DES packages in each language: SimPy ( Team SimPy, 2024) for Python and simmer ( Ucar et al., 2019) for R. Both are widely used, actively maintained and extensively tested. Dependencies were managed using Conda for Python and renv for R.

The e-book is built with Quarto ( Allaire et al., 2024) as this allows narrative text/markdown and executable Python and R code to co-exist in the same version-controlled document. The website is hosted freely on GitHub Pages and rendered using GitHub Actions. It builds the site inside a Docker container, which is defined by the environment specifications and stored in the GitHub Container Registry, reused or updated for subsequent builds or deployments.

All resources were developed using version control (Git) and are hosted on GitHub. All code is openly licensed under MIT and text under CC-BY-SA 4.0. To guarantee long-term accessibility, automated workflows deposit each major release to Zenodo, providing a permanent digital object identifier (DOI) for that version of the resource.

The online book (and four case studies below) was designed to address all items in: (1) the reproducibility recommendations from Heather et al. (2025) (Appendix A), and (2) the *Levels of RAP* from NHS RAP Community of Practice (2025a) (Appendix B).

### 3.2 FAIR design of the book

Many of the design choices described above were made explicitly to ensure training materials were FAIR (Findable, Accessible, Interoperable and Reusable). To further ensure the content and infrastructure of the book align with FAIR principles, we used an in-development checklist from the digital Research Technical Professionals (dRTP) Skills CHARTED (Connecting Hub for Advancing the RTP Talent Enabling DRI) project to evaluate FAIRness of training resources ( dRTP Skills, 2026). This checklist adapts questions from existing FAIR training guidance ( Garcia et al. (2020), Software Sustainability Institute (SSI) (2026)). For example, ensuring there are clear learning outcomes and descriptions of the intended audience, case studies illustrating use, and estimates of time commitment; offering opportunities for learners to feedback and obtain support; supplying preparatory instructions; registering the resource in training registries; embedding machine-readable metadata; and following web accessibility conventions and standards. The book was developed by a single researcher, then underwent iterative peer review by PhD students and subject matter experts on the project team.

Each section of the book provides parallel implementations in both Python and R, with an interactive toggle allowing readers to switch between languages. This approach ensures learners can follow examples in their preferred language while also observing how core concepts translate across ecosystems. Interactive features include hover-based code comparisons that show incremental changes as the book progresses. An example screenshot from the e-book is provided in Figure 2.

**Figure 2.**
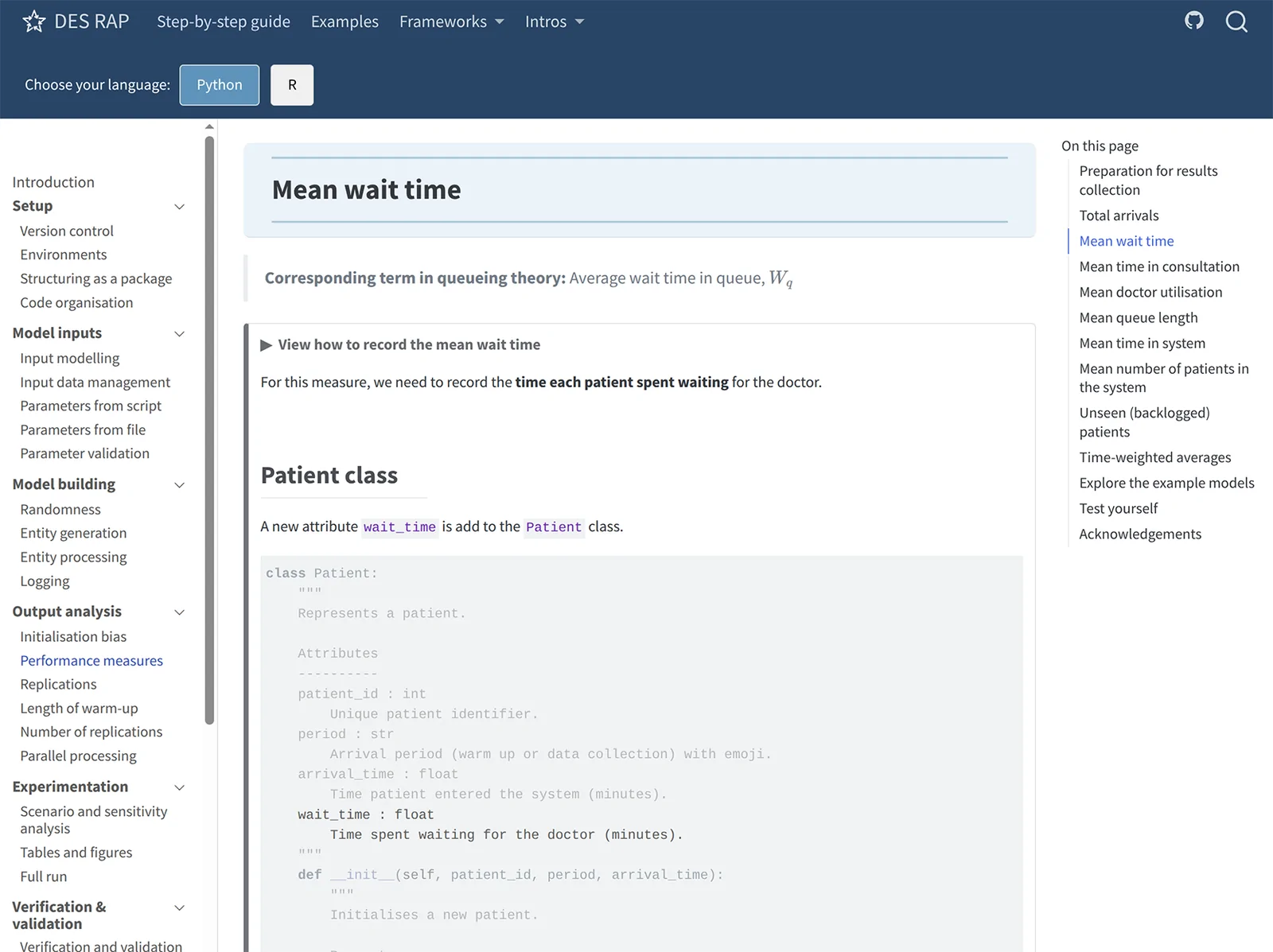
E-book screenshot.

### 3.3. Contents of the online book

**3.3.1. Introductory material**

The introductory chapters establish a shared baseline before learners start building models. The DES introduction uses simple queueing examples and animations (created using the vidigi package ( Rosser and chalk, 2026)) to show why DES is useful in healthcare, and to establish terminology for entities, resources, queues, activities/processes and events. It also introduces common modelling styles (activity-based, event-based, process-based and three-phase) and explains how stochasticity is handled via sampling from probability distributions, so that later code examples can be read in terms of standard DES concepts.

A separate introduction page defines reproducibility and reproducible analytical pipelines (RAPs), and explains why these ideas matter for simulation studies. A third page explains why the book is built around free and open-source software (FOSS), clarifying what is meant by FOSS and outlining why it is often recommended for RAPs: no license barriers, no restrictions on distribution, no risk of losing access, and greater transparency. Finally, two framework pages situate the book within existing guidance by mapping chapters to the recommendations of Heather et al. (2025) and to the NHS “Levels of RAP” maturity framework ( NHS RAP Community of Practice, 2025b).

**3.3.2. Set-up**

The set-up chapters focus on the practical scaffolding needed for a reproducible DES RAP project before any modelling code is written. The version control chapter introduces Git and GitHub, and shows how to initialise a repository, commit changes, and use branches to manage development. It emphasises keeping all model, analysis and documentation files under version control so that changes are tracked and specific versions of the project can be recovered when needed.

A second chapter covers reproducible environments, demonstrating how to create and share isolated environments for both Python and R. A third chapter explains how to set up a basic package structure for the project, outlining the benefits for reuse and testing. Finally, a chapter on code organisation introduces principles of modular code and shows how to write functions and classes, for readers who may not have encountered these patterns before.

**3.3.3. Model inputs**

The model inputs section focuses on how to specify, manage and validate the parameters used in a DES study. The input modelling chapter guides learners through how to inspect, fit and select probability distributions using both targeted (candidate distributions) and comprehensive approaches. It makes use of the distfit package in Python ( Taskesen, 2025) and fitdistrplus in R ( Delignette-Muller and Dutang, 2015).

A second chapter addresses input data management, clarifying where a RAP begins and introducing recommended practices for storing and sharing raw data, input modelling code and fitted parameters, and discusses how to handle sensitive data. Two chapters then focus on parameter handling. The first explains the drawbacks of hard-coded parameters, presents strategies for organising large sets of parameters in scripts, and motivates importing parameters from external files. The second shows how to create and document such parameter files (including data dictionaries) and how to load them into Python and R. Finally, a parameter validation chapter introduces simple checks to prevent accidental creation of new parameters and to ensure parameters fall within expected ranges, supporting more robust and transparent model configuration.

**3.3.4. Model building**

The model building chapters introduce how randomness and process logic are implemented in code. The first chapter explains pseudorandom number generators and the tools used for random sampling in each language, including NumPy ( Harris et al., 2020) and sim-tools ( Monks et al., 2026) in Python, and the core stats package and simEd ( Lawson et al., 2025) in R. Learners are shown how to control seeds, draw samples from common distributions, and are taught about independent number random streams.

Subsequent chapters construct a simple DES model step by step. In Python, the model is built using an object-oriented approach with classes, while in R it is implemented using functions; in both cases the structure mirrors the layout later used in the worked examples. The initial model includes only arrivals and resource use, with each new function or class introduced incrementally and its role explained. A final chapter covers model logging and tracing. In Python, this begins with simple print statements and then shows a small logging class built on the standard logging module. In R, learners are shown how to interpret simmer’s default logs and how to create custom logs using attributes.

**Figure 3.**
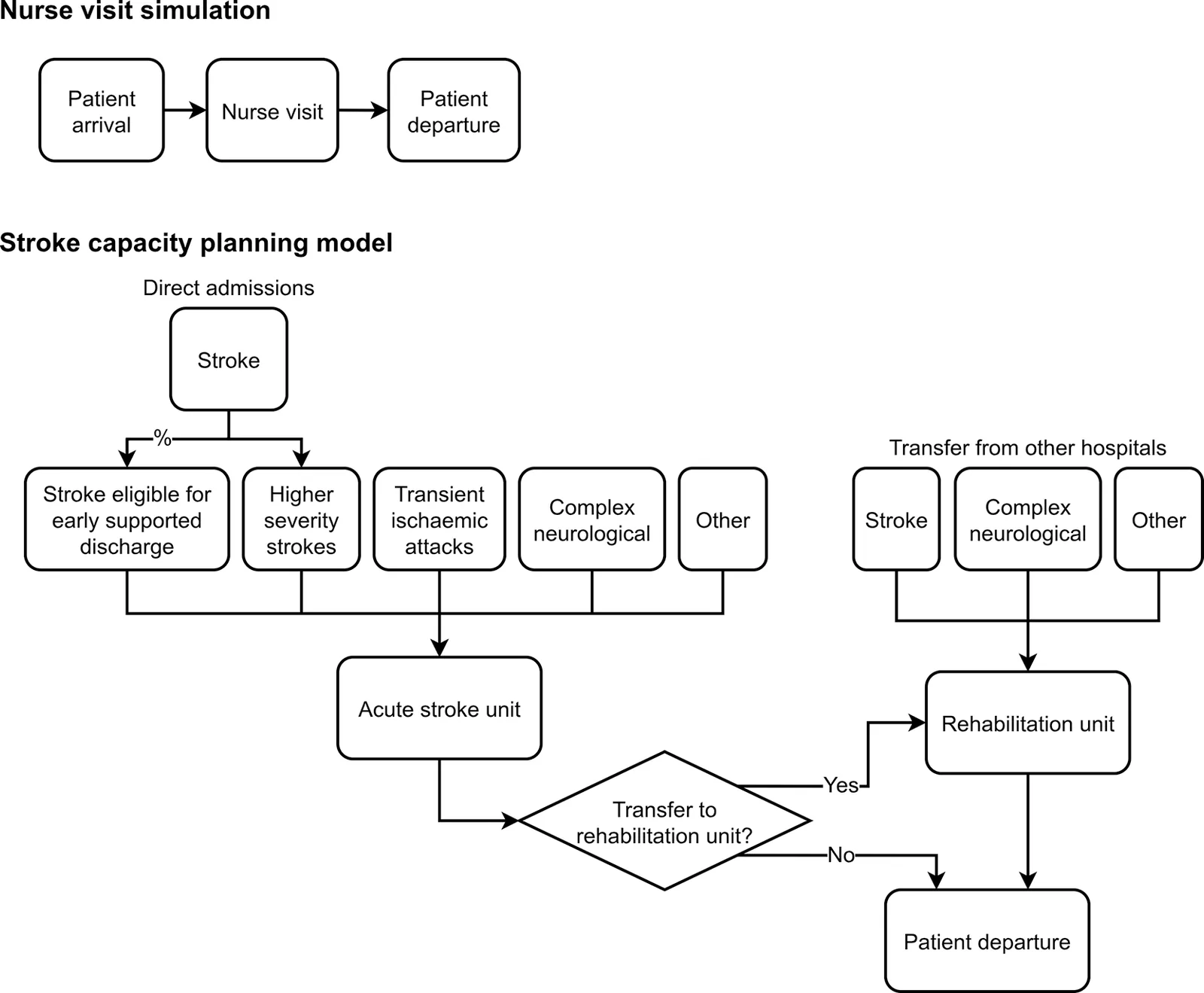
Structure of the nurse visit simulation and stroke capacity planning model.

**Figure 4.**
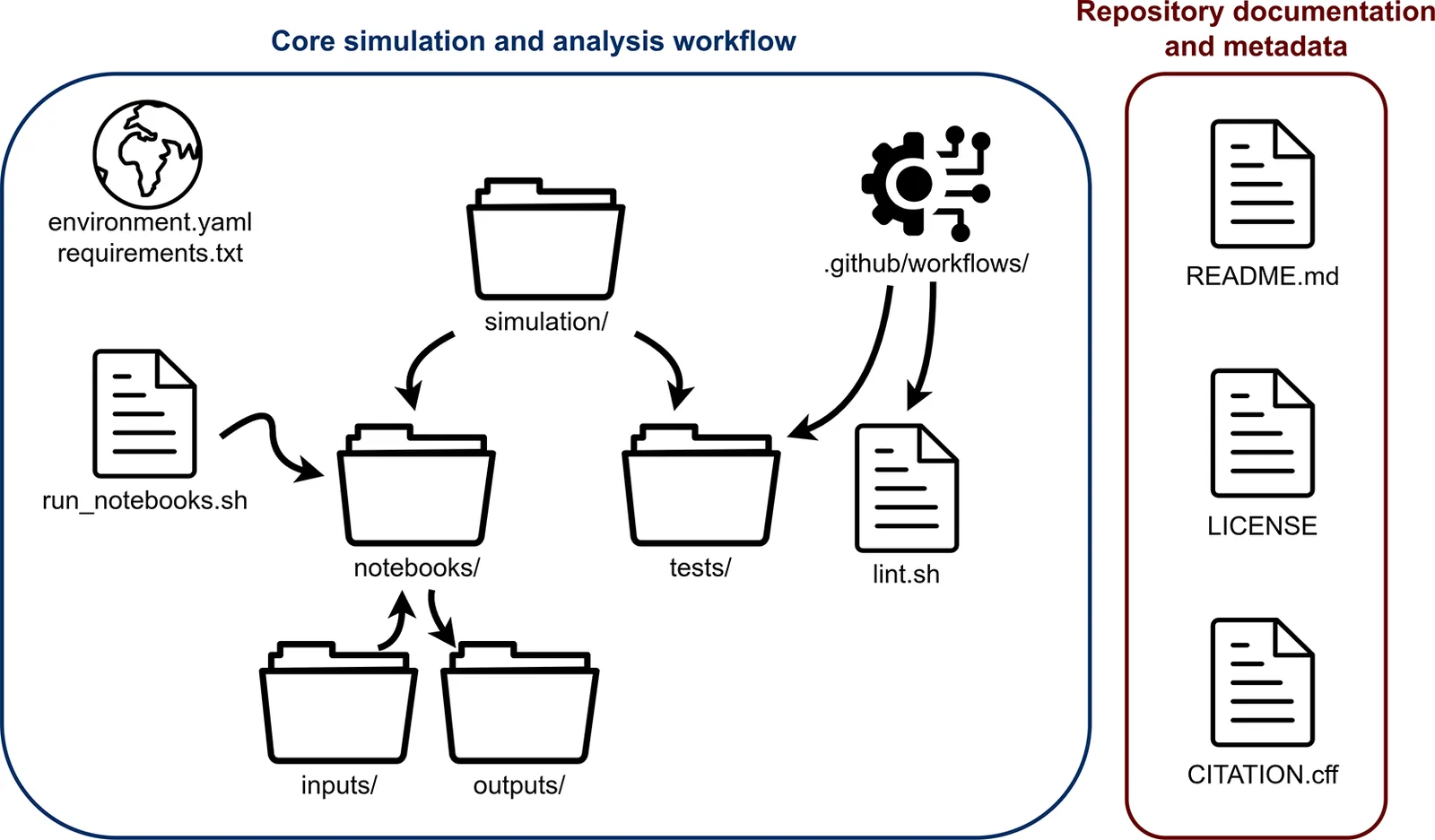
Key workflows and files from the Python repositories - R repositories followed similar equivalent structure.

**Figure 5.**
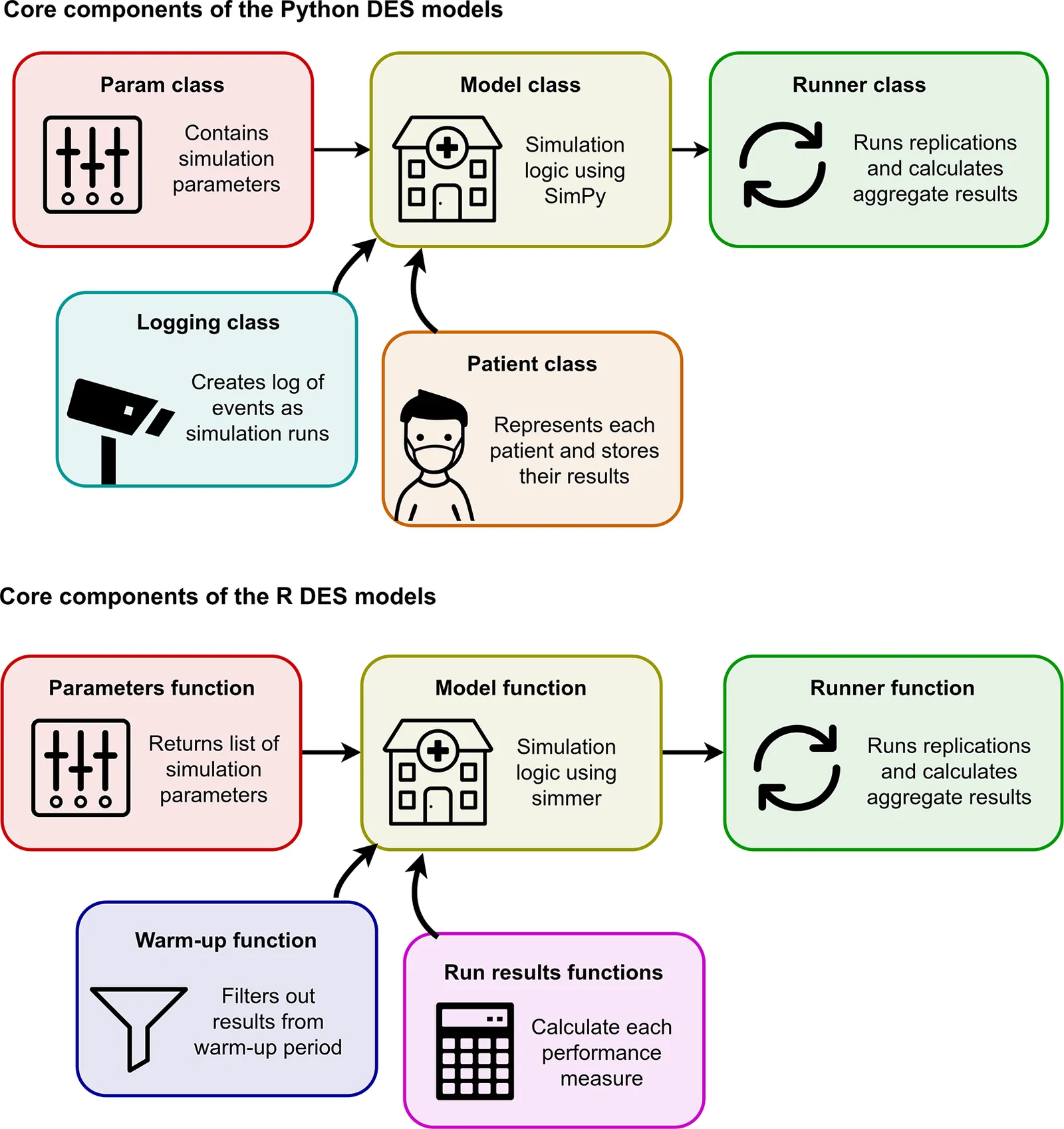
Core components of modular Python and R DES codebase.

**3.3.5. Output analysis**

The output analysis section covers how to obtain reliable performance measures from the simulation runs. The initialisation bias chapter explains why starting from an empty system can distort early results and introduces warm-up periods. These are implemented in Python by resetting the results-collection lists once the warm-up period has elapsed during the SimPy run, whereas in R with simmer the warm-up is handled after the run by filtering the monitored output to remove data from the warm-up window.

A second chapter shows how to record key performance measures. These measures and their corresponding terms in queueing theory are summarised in Table 1. The subsequent chapters explain how to run multiple replications and how to choose how many to run using a confidence-interval-based method, as well as how to select an appropriate warm-up length using time-series inspection. These methods follow the recommendations of Robinson (2025) and use the replications algorithm from Hoad et al. (2010). The final chapter demonstrates how to run replications in parallel and how to choose an appropriate number of cores.

**Table 1.** Performance measures covered in the book and their corresponding queueing-theory notation.

Performance measure	Corresponding term in queueing theory
Total arrivals	N/A
Mean wait time	Average wait time in queue, $W_{q}$
Mean time with resource	Service time, 1/μ
Mean resource utilisation	Server utilisation, ρ
Mean queue length	Average number in queue, $L_{q}$
Mean time in system	Average time in system, W
Mean number of patients in the system	Mean system size or average number in the system, L
Backlogged patient count and mean wait time	N/A

**3.3.6. Experimentation**

The experimentation chapters show how to structure and share analysis of the model results. The scenario and sensitivity analysis chapter explains the difference between scenario analysis and sensitivity analysis, and provides functions in both Python and R to generate and run all combinations of scenarios programmatically rather than by hand. It emphasises that the code used to define and run these analyses should be shared alongside the model.

A second chapter focuses on tables and figures, highlighting the importance of sharing the code used to create summary tables and plots rather than only the outputs, and illustrating this with example scripts for common DES performance measures. The final chapter in this section argues that a RAP should be runnable from start to finish with a single command. It demonstrates how to chain multiple scripts or notebooks so that the complete workflow (from data and inputs through simulation runs, scenario analyses, and output figures) can be re-executed and audited as a single, end-to-end process.

**3.3.7. Verification and validation**

The verification and validation (V&V) chapter introduces key V&V concepts from the simulation literature (for example, many of those outlined by Balci (1998)). For each concept, it explains what they mean in practice for a DES RAP project. This is provided as a checklist (Appendix D) with actionable activities for V&V, and a Markdown version is supplied in the online book so it can be copied into a GitHub issue and used to track V&V work openly. Following this structured and documented approach to V&V aligns with TRACE (TRAnsparent and Comprehensive Ecological modelling documentation), which frames these activities as part of a broader, planned and documented process for establishing model quality and credibility across the whole model lifecycle ( Grimm et al., 2014).

It then shows how to write and run tests for simulation models using pytest ( Krekel et al., 2004) and testthat ( Wickham, 2011), before describing how to verify parts of a model using mathematical proofs of correctness by comparing analytical results with those from the simulation. Finally, a quality assurance chapter explains what QA means in this context, how to plan QA activities using UK government guidance such as the AQuA Book ( Government Operational Research Service et al., 2025), and how to maintain a transparent QA plan and log (for example, with GitHub Projects) across the whole project lifecycle to support trust in the model and its results.

**3.3.8. Style and documentation**

This section begins with a chapter on style guides, linters and formatters, and demonstrates how to configure and run several common tools. A subsequent chapter covers docstrings, explaining why in-code documentation matters for reuse and maintenance and giving concrete patterns for writing effective docstrings in both Python and R. The section then introduces continuous integration with GitHub Actions, outlining how continuous integration and continuous delivery/deployment (CI/CD) supports RAP workflows and walking through simple workflows that run tests, calculate test coverage, and run linters. Finally, a documentation chapter distinguishes different forms of project documentation, clarifies what core files like README.md and CONTRIBUTING.md should contain, and illustrates options for generating documentation websites.

**3.3.9. Collaboration and sharing**

The first chapter covers code review: the purpose of review, when to use it, which tools and platforms to use, who should be involved, and what reviewers should look for in simulation code and documentation. Subsequent chapters cover licensing, outlining why software and text need explicit licences, how to choose an appropriate licence, and how to add it correctly to a repository, and citation, which explains why clear citation instructions matter and shows how to provide them (for example with a CITATION file or CITATION.cff) so others can acknowledge and reuse your work properly. The section then introduces changelogs and semantic versioning, describing how to record changes over time, use version numbers consistently, and create GitHub releases. Finally, an archiving chapter shows how to share simulation code in the long term, summarising reporting and sharing guidelines and demonstrating how to archive a repository on Zenodo via GitHub releases so that each version has a persistent DOI.

## 4. Four worked example repositories

### 4.1. Model case studies

The example repositories demonstrate RAP in practice using two simulation cases of differing complexity. The first is a model with simple structure and entirely synthetic inputs. The second is a real-world model that replicates a published stroke pathway simulation of a healthcare system using empirically derived inputs.

**Synthetic case: nurse visit simulation**. This is a simple model of patients arriving and waiting for a nurse visit, and is structured as an M/M/s queueing model. The M/M/s queue is a classic queueing model which meets the Markovian assumptions of having Poisson arrivals (M) and exponential service times (M), and then s identical servers sharing a single queue (s). It requires only three parameters: arrival rate, service rate and number of servers ( Green, 2011). As it follows a simple structure, and is constructed with just synthetic data, this model allows learners to focus entirely on the core principles of building DES in RAP (e.g., implementing a basic DES, specifying inputs, selecting warm-up and replications, analysing standard performance measures), without the distraction of complex clinical pathways or inputs.

**Real-world case: stroke pathway model**. To demonstrate RAP in a real-world model, we replicated the stroke care pathway model originally developed in Simul8 by Monks et al. (2016). This model involves multiple patient types, more complex routing logic, and extensive parameter sets. We knew successful replication was feasible as all parameters and logic were fully specified in the original publication, as verified in prior work ( Monks et al., 2025).

The structure of the models is illustrated in Figure 3, with further details on each model provided via STRESS-DES checklists in Appendix C.

### 4.2. Repository structure

As shown in Figure 4, each repository is structured as a local package, with the simulation code placed in its own package directory and accompanied by a dedicated test suite. This separates reusable simulation logic from notebooks, scripts, and documentation, and follows good practice for keeping model code modular and easier to understand, test, and maintain.

The root folder contains standard project files: a README with set-up and run instructions, LICENCE and CITATION.cff files, contribution guidelines, and a code of conduct. Dependency management is handled via environment.yaml and requirements.txt for Python, and DESCRIPTION together with renv.lock for R, so that users can recreate the analysis environment consistently. Bash scripts (for example, run_notebooks.sh) provide a single entry point to execute the full workflow, running all analyses and regenerating outputs.

Automated checks are implemented using GitHub Actions workflows. These workflows install the project, recreate the environment, and then run the test suite and linters when changes are pushed. This helps ensure that both the Python and R repositories remain runnable, tested, and style-compliant over time.

### 4.3. Code structure

Within the package, the simulation code is organised into a small number of focused components, as illustrated in Figure 5. For example, separate classes or functions are used for setting up parameters, implementing the core simulation logic, and running replications and experiments. This modular structure makes the code easier to navigate, test, and adapt, because each part has a clear responsibility.

Across both languages, code style follows established community standards: PEP-8 with NumPy-style docstrings in Python, and tidyverse style with roxygen2 docstrings in R ( van Rossum et al., 2001; numpydoc maintainers, 2026; The tidyverse team, 2025; Wickham et al., 2025). Linters such as pylint, flake8, nbqa, and lintr are used to check adherence to these styles, supporting readability, peer review, and long-term maintainability ( Pylint contributors, 2026; flake8 contributors, 2025; nbQA contributors, 2026; Hester et al., 2025).

### 4.4. Verification and validation

Verification involves checking that the simulation model correctly implements the intended conceptual model. We ensured this by implementing several verification strategies from Balci (1998):

• **Desk checking** - Included code peer review and linting.
• **Debugging** - Bugs were recorded and tracked using GitHub Issues. The models were supported by automated unit and functional test suites that were expanded as new bugs were found and fixed. Tests were developed using pytest in Python ( Krekel et al., 2004) and testthat in R ( Wickham, 2011).
• **Assertion checking** - Assertions are explicit statements that outline expected model behaviour (e.g., patient flow logic, resource constraints, parameter validation). These assertions were implemented within the model code and via tests to flag conditions that appeared incorrect or unexpected.
• **Special input testing** - This included stress tests (to simulate heavy demand) and idle system tests (to simulate scenarios with little or no activity, waiting, or service).
• **Bottom-up testing** - Unit tests were written for individual model components, and functional tests were developed to verify the combined behavior of integrated components.
• **Regression testing** - Tests were developed early in model development and run regularly as part of a continuous integration pipeline. All tests were re-run upon merges into the main branch via GitHub Actions. Back tests were included to ensure consistency of results over time.
• **Mathematical proof of correctness (for M/M/s model only)** - Simulation results were compared against theoretical queueing solutions derived analytically from mathematical equations.

Validation involves checking whether the simulation model is a sufficiently accurate representation of your real system. In our case, this was limited as the M/M/s examples used synthetic inputs and the stroke model simply replicated an existing model with no intention of reapplying and needing to check it for a new clinical setting. However, there was one validation felt suitable to perform: **comparison testing**. As described in Balci (1998), this involves comparing multiple simulation models of the same system. In this case, results from Python and R models were compared. For the stroke model, these were also compared against the reported results from the original Simul8 implementation Monks et al. (2016).

Overall, both verification and validation indicated that the implemented models behaved as intended and produced results consistent with theoretical and prior implementations.

## 5. Accessing the resources

The online book and all four example repositories are openly available under MIT software licences, with archived versions assigned DOIs. Links to the resources are provided in the software availability statement.

## 6. Discussion

### 6.1. Overview of contributions

This paper presents an open, executable methodological guide and accompanying case studies that are designed to bridge the gap between reproducibility principles and day-to-day practice in healthcare DES. Together, the guide and four worked example repositories provide what no existing resource offers: integrated RAP workflows for DES in both Python and R that turn abstract recommendations into concrete, fully implemented examples.

The worked examples serve dual purposes: they demonstrate that RAP principles are achievable for healthcare DES models of varying complexity (from canonical queueing systems to real-world clinical pathways), and they provide templates that modelling teams can use as starting points for their own work.

Our contribution is not the individual practices themselves, as things like version control, modular code structure, testing, or dependency management are all well-established. Instead, it is their integration into complete, end-to-end workflows for healthcare DES in both Python and R. By showing how the STARS reproducibility recommendations and NHS Levels of RAP can be realised in concrete DES models, the resources move from abstract guidance to operational practice. Although the case studies are drawn from healthcare, the underlying processes are relevant to DES more broadly and can also serve as a general resource on implementing RAP in simulation studies. Within healthcare organisations, they offer a concrete blueprint for teams seeking to standardise their simulation workflows and meet emerging expectations around transparency and reproducibility. This is particularly important for long-lived operational DES models in health services, where the same models may underpin multiple business cases, service reviews, and updates over several years.

### 6.2. Dual-language design

The decision to provide parallel implementations in Python and R reflects the reality that both languages are widely used in healthcare simulation research ( Monks and Harper, 2023). In addition, using R aligns our examples with current practice in health economic modelling, where R is now the most common platform for open-source models ( Henderson et al., 2025). Rather than advocating for one language over another, we aimed to support both communities while enabling cross-language comparison and learning. This dual-language approach required significantly more development effort but provides unique value: modelling teams can see how the same concepts are expressed in different ecosystems, teams can choose the language that best fits their existing workflows and expertise, and the act of implementing the same model in two languages serves as a form of validation ( Balci, 1998), ensuring that the underlying simulation logic is correctly specified and language-independent. The selection of SimPy and simmer as the simulation libraries, rather than implementing DES engines from scratch, reflects software engineering best practice: reusing well-tested, community-supported libraries reduces errors, improves maintainability, and allows focus on model logic rather than simulation mechanics.

In practice, we found no meaningful differences in performance or ease of development between Python and R, with the best choice of language for modellers depending on team expertise, existing codebases, and personal preference. By presenting solutions in both, we aim to support use of either language.

### 6.3. Ease of use

A likely concern is whether healthcare modelling teams will realistically adopt workflows of this scope. The resources were designed deliberately to make this as easy as possible. The book does not just present finished models; it walks step by step from basic concepts to full RAP implementations, so that readers can build the required understanding as they go. The worked repositories follow the same path and can be used as informal templates: modelling teams can copy their structure and patterns, adopt core elements first (such as code structure and documentation), and only later introduce components like packaging, testing or continuous integration.

### 6.4. Practical applications

These resources have several practical applications:

• **For modelling and analytics teams in healthcare organisations like the NHS:** concrete RAP patterns they can adopt to improve transparency, trust, and collaboration when DES is used for operational and strategic decision-support.
• **For learners new to DES:** a step-by-step guide to DES modelling while embedding RAP practices from the outset.
• **For experienced DES modellers:** clear examples of how to apply RAP principles in healthcare DES, supporting those looking to make their work more transparent and reproducible.
• **For anyone publishing DES results in journals or conferences:** following a RAP workflow will meet emerging reproducibility requirements, including initiatives such as the Journal of Simulation’s Model Reproducibility Initiative (JOS–MRI) ( Operational Research Society, 2025) and artefact review and reproducibility badging schemes from the Association for Computing Machinery (ACM) (2020).
• **For educators:** openly licensed materials, available for use within teaching courses on DES, RAP, or reproducible research in healthcare.
• **For healthcare organisations and institutions:** practical examples of “gold tier” reproducibility standards, helping establish standards and expectations for high-quality and reproducible DES work.

### 6.5. Openness and sustainability

Open licensing (MIT for code, CC-BY-SA 4.0 for text) and public archiving via Zenodo with DOIs ensure the resources remain freely accessible and citable. The version-controlled GitHub repositories enable community contributions, corrections, and extensions, allowing the resources to evolve as users identify gaps or opportunities for improvement. Containerisation and automated Docker builds for the online book help ensure the materials remain executable and reproducible over time.

### 6.6. Limitations

The resources cover Python and R as the most widely used languages for healthcare DES ( Monks and Harper, 2023), but do not include other possible languages used in simulation research like Julia and C++. Modelling teams working in other languages will need to adapt the principles described here rather than directly reusing the code.

They also do not address commercial or proprietary software platforms. These platforms have historically dominated healthcare DES use, with over 60% of studies using commercial software, with ARENA, AnyLogic and Simul8 most common ( Monks and Harper, 2023; Vázquez-Serrano et al., 2021). While graphical interfaces and vendor support make these tools feel more accessible in some settings, they also create barriers to reproducibility: licences are expensive, terms often restrict code sharing, and models cannot be inspected or reused without access to the same software. Although some RAP practices can be applied within these environments, closed-source architectures and licensing constraints limit the openness, sharing, and long-term archiving that RAP aims to support. Our focus on free and open-source tools is therefore a deliberate choice to prioritise reproducibility and equity.

## 7. Conclusion

This paper provides the first comprehensive set of open-source resources demonstrating RAP workflows for healthcare DES in both Python and R. By grounding practical guidance in full worked examples verified through code review, automated testing, and cross-language comparison, we address a key barrier to RAP adoption: the lack of accessible, complete demonstrations of how to implement reproducibility in practice. By emphasising modular design, version control, and automated workflows, the resources also support more sustainable operational use of DES models, making it easier to maintain, update, and safely reuse them within healthcare organisations. All materials will remain freely accessible and versioned via GitHub and Zenodo, supporting long-term reuse and citation.

However, the resources cannot address systemic barriers to reproducibility: institutional cultures that undervalue code sharing, publication policies lacking reproducibility requirements, and time pressures discouraging reproducibility investment. Policy changes (e.g., journal requirements and awards for open and reproducible code) can help amplify the impact of training resources like ours. Similarly, organisational commitment to dedicating time and support for implementing reproducible practices is essential.

## Software availability statement

All code, simulation models and materials for this study are openly available in the following repositories and archives.

**Online book:**

• Website: https://pythonhealthdatascience.github.io/des_rap_book/
• Source code available from: https://github.com/pythonhealthdatascience/des_rap_book

• Archived software available from: https://doi.org/10.5281/zenodo.17094155
• Citation: Heather et al. (2026b)
• License: MIT and CC-BY-SA 4.0

**Python M/M/s model:**

• Source code available from: https://github.com/pythonhealthdatascience/pydesrap_mms
• Archived software available from: https://doi.org/10.5281/zenodo.14622466
• Citation: Heather and Monks (2026a)
• License: MIT

**R M/M/s model:**

• Source code available from: https://github.com/pythonhealthdatascience/rdesrap_mms
• Archived software available from: https://doi.org/10.5281/zenodo.14980863
• Citation: Heather and Monks (2026b)
• License: MIT

**Python stroke model:**

• Source code available from: https://github.com/pythonhealthdatascience/pydesrap_stroke
• Archived software available from: https://doi.org/10.5281/zenodo.15574906
• Citation: Heather and Monks (2026c)
• License: MIT

**R stroke model:**

• Source code available from: https://github.com/pythonhealthdatascience/rdesrap_stroke
• Archived software available from: https://doi.org/10.5281/zenodo.15863376
• Citation: Heather and Monks (2026d)
• License: MIT

## Data Availability

This study uses only synthetic or previously published parameters, as described in Appendix C. These parameters can be accessed within the repositories linked in the software availability statement. The study appendices are available as supplementary materials hosted on OSF under a CC-BY license at https://doi.org/10.17605/OSF.IO/ZN8P5 ( Heather et al., 2026a). These include:
• Appendix A: STARS Reproducibility Recommendations. • Appendix B: NHS England RAP Community of Practice “Levels of RAP”. • Appendix C: STRESS-DES checklists. • Appendix D: Verification and validation checklist.
