# Supplementary material for: Reproducible analytical pipelines for healthcare discrete‑event simulation: An open guide and worked examples
Source: NIHR Open Res. 2026 Jun 16;6:68. [Version 1] doi: 10.3310/nihropenres.14296.1 (PMC13675625; doi:10.3310/nihropenres.14296.1)
Supplement: Peer review history

# Reviewer report: Reviewer response for version 1

**DOI:** 10.3310/nihropenres.15573.r42502

**Published:** 2026-10-16

**Authors:** Osama Alotaik

**Affiliations:** 1 King Saud University, Riyadh, Saudi Arabia

This article presents an open online book and four worked example repositories that show how to build healthcare discrete-event simulation (DES) models as reproducible analytical pipelines (RAPs) in Python and R. The article is clearly written, and the resources it describes are of practical value. In fact, although the case studies come from healthcare, most of the guidance applies directly to DES studies in other application areas, e.g., manufacturing and logistics. However, I would like the authors to consider my minor suggestions given below:

 1- Section 1.3 discusses the reproducibility of published simulation studies, but it does not mention that the dominant tools in simulation practice are commercial, GUI-based packages such as Arena, AnyLogic, and Simio. The authors raise this point only briefly at the end of the article (Section 6.6). There they note that over 60% of studies use commercial software and that graphical interfaces can make these tools more accessible. I believe this point deserves elaboration in the main text. Many modelers use GUI tools even when they have a good background in Python or R, because these tools are more accessible and make visualization easy, e.g., 3D animation of the model and built-in plots of the results. Visualization is essential here, since it makes it much easier to communicate a model and its results to non-technical audiences. So, a reader coming from a GUI tool will likely ask: what do I give up by moving to a code-based RAP, and how much of it can I recover? A short discussion of this trade-off in Section 1.3 would make the case for RAPs more convincing to this group of modelers. It could also point to the open-source options already in the book, namely the vidigi package used for the introductory animations and the “Tables and figures” chapter.

 2- AI assistance as a future direction. This is a suggestion rather than a concern about the article. The accessibility issue in point 1 above is one reason modelers do not adopt open-source tools in the first place, even when they have a programming background. A reasonable extension of this work is to develop AI-assistant functionality around the book and the example repositories. For example, an assistant could help a modeler turn a conceptual model into code that follows the book’s structure, or produce the tests and plots the book describes. The authors’ prior work on using generative AI to reconstruct healthcare simulation models (Monks et al., 2025) suggests they are well placed to pursue this, and a sentence on it in the Discussion would be a useful addition.

Are the conclusions about the tool and its performance adequately supported by the findings presented in the article?

Yes

Is the rationale for developing the new software tool clearly explained?

Yes

Is the description of the software tool technically sound?

Yes

Are sufficient details of the code, methods and analysis (if applicable) provided to allow replication of the software development and its use by others?

Yes

Is sufficient information provided to allow interpretation of the expected output datasets and any results generated using the tool?

Yes

Reviewer Expertise:

Operations Research, Machine Learning, and Simulation Modeling in logistics, healthcare, and manufacturing.

I confirm that I have read this submission and believe that I have an appropriate level of expertise to confirm that it is of an acceptable scientific standard.

# Reviewer report: Reviewer response for version 1

**DOI:** 10.3310/nihropenres.15573.r42680

**Published:** 2026-10-15

**Authors:** Adrián Borbolla-Muñoz

**Affiliations:** 1 Technical University of Denmark, Kongens Lyngby, Denmark

The authors present a comprehensive set of resources integrating Reproducible Analytical Pipeline (RAP) into open-source healthcare DES models to facilitate reproducibility and replicability. They provide an open online book, with an educational focus, which include a step-by-step approach and two case study applications using two different programming languages. This paper makes a meaningful contribution in the ongoing issue of reproducibility and good reporting practices of DES models. The concepts discussed are very relevant and are presented in a practical way that can be understood and applied by both beginners and skilled modellers. There are a few points that could be improved to make some aspects clearer and more complete. My suggestions are:

- Minor corrections:
  - Some acronyms appear before being introduced or defined in the paper, such as " *NHS"* or *"QA"*.
  - I noticed a minor punctuation issue at Section 1.3, paragraph 5, just after the citation to Henderson et al., there appears to be missing a full stop. I recommend the authors to check the paper for similar minor issues (if there are).

 

- In the introduction, the authors provide an overview of previous work aiming to improve reproducibility in healthcare DES models (e.g., STRESS and STARS). However, the relationship between previous work and the proposed RAP workflow is unclear. This part could be expanded by clarifying the limitations of existing approaches and how RAP addresses or complements them. Moreover, I would encourage the authors to provide some context explaining how previous work has influenced the design and formulation of the proposed solution.

 

- The authors state that they provide the first comprehensive set of open-source resources demonstrating RAP workflows for healthcare DES. It would be interesting to understand what adaptations were necessary from traditional RAP practices to specifically address healthcare DES applications. Also, I believe that this work has the potential to be generalised to other DES domains. Although discussed shortly in the discussion section, it would be valuable to expand a bit more on what future work could be done to enable this.

 

- Section 3 presents the concepts implemented in the online book. Although the subsections are clearly explained, I suggest adding a high-level figure that summarizes the overall workflow and the major concepts covered in this section. This will provide a quick overview of the proposed step-by-step workflow and help the reader to navigate through the paper and book.

 

- The paper acknowledges that over 60% of the healthcare DES studies use commercial software. Although the authors clearly justify their focus on open source as a means of enhancing reproducibility, it would be valuable to discuss how they foresee reproducibility challenges in DES evolving over time and the long-term adaptations and applicability of the presented work

**AI Disclosure Statement:** Copilot, GPT-5 model was used only to assist with grammar, and orthography checks of author-generated text. All comments and interpretations were developed by the reviewer who takes full responsibility for the report.

Are the conclusions about the tool and its performance adequately supported by the findings presented in the article?

Yes

Is the rationale for developing the new software tool clearly explained?

Partly

Is the description of the software tool technically sound?

Yes

Are sufficient details of the code, methods and analysis (if applicable) provided to allow replication of the software development and its use by others?

Yes

Is sufficient information provided to allow interpretation of the expected output datasets and any results generated using the tool?

Yes

Reviewer Expertise:

Integration of discrete event simulation and life cycle assessment for sustainability transition assessments and decision support in manufacturing systems.

I confirm that I have read this submission and believe that I have an appropriate level of expertise to confirm that it is of an acceptable scientific standard, however I have significant reservations, as outlined above.

# Reviewer report: Reviewer response for version 1

**DOI:** 10.3310/nihropenres.15573.r41350

**Published:** 2026-08-22

**Authors:** S M Atikur Rahman

**Affiliations:** 1 University of Texas at El Paso, El Paso, TX, USA

This manuscript presents a reproducible analytical pipeline based on discrete-event simulation (DES) for healthcare applications. The authors clearly describe the workflow, and the inclusion of open-source code through GitHub significantly improves the reproducibility and transparency of the study. The case studies, including an M/M/s queueing model and a stroke care pathway model, demonstrate the applicability of the proposed approach across different healthcare scenarios. Overall, the manuscript is well structured and provides a valuable contribution. However, the following minor suggestions could further strengthen the work:

 1. It would be good if the author showed the specific queue model with an example in real settings, as the real queue system in a hospital is quite tough. 

 2. Sensitivity analysis can be performed to strengthen the model validity in statistical settings. 

 3. Benchmarking Python and R with the established simulation process, for example, FlexSim, AnyLogic, and Arena, would further strengthen the case study for visualization purposes (though it is mentioned in the practical application section).

Are the conclusions about the tool and its performance adequately supported by the findings presented in the article?

Yes

Is the rationale for developing the new software tool clearly explained?

Yes

Is the description of the software tool technically sound?

Yes

Are sufficient details of the code, methods and analysis (if applicable) provided to allow replication of the software development and its use by others?

Yes

Is sufficient information provided to allow interpretation of the expected output datasets and any results generated using the tool?

Yes

Reviewer Expertise:

Simulation and Machine learning in healthcare, SCM, and manufacturing.

I confirm that I have read this submission and believe that I have an appropriate level of expertise to confirm that it is of an acceptable scientific standard.
